# Structured approaches to promote patient and family engagement in treatment in acute care hospital settings: protocol for a systematic scoping review

**DOI:** 10.1186/s13643-018-0694-9

**Published:** 2018-02-26

**Authors:** Donna Goodridge, Chrysanthus Henry, Erin Watson, Meghan McDonald, Lucia New, Elizabeth L. Harrison, Murray Scharf, Erika Penz, Steve Campbell, Thomas Rotter

**Affiliations:** 1 College of Medicine, University of Saskatchewan, Saskatoon, Canada; 2 Department of Community and Population Health Studies, College of Medicine, University of Saskatchewan, Saskatoon, Canada; 3 Leslie and Irene Dube Health Sciences Library, University of Saskatchewan, Saskatoon, Canada; 4 College of Medicine, Health Sciences Graduate Program, University of Saskatchewan and Saskatchewan Collaborative Bachelor of Science in Nursing Program, Saskatchewan Polytechnic, Saskatoon, Canada; 5 School of Physical Therapy, College of Medicine, University of Saskatchewan, Saskatoon, Canada; 6 College of Education, University of Saskatchewan, Saskatoon, Canada; 7 Faculty of Health, University of Tasmania, Hobart, Australia; 8 Healthcare Quality Programs, Queen’s University, Kingston, Canada

**Keywords:** Patient and family engagement, Patient and family-centered care: behavior change techniques, Acute care hospitals

## Abstract

**Background:**

While effective engagement of patients and families in treatment is increasingly viewed as a priority for many healthcare systems, much remains to be learned about the nature and outcomes of approaches that seek to accomplish this goal in the acute care hospital setting. Wide variability in the implementation of practices designed to promote patient and family engagement in hospitals has been noted. Approaches aimed at promoting patient and family engagement in treatment share the over-arching goal of changing behaviors of patients, families, and healthcare providers and possibly administrators. Behavior change techniques (BCTs) can be a key element of patient and family engagement approaches. This scoping review will contribute to the development of an evidence base detailing that the BCTs have potential to be effective in patient and family engagement interventions. The specific objectives of this review are to (a) identify and classify approaches used in acute care hospitals to engage patient and families in treatment according to the behavior change technique taxonomy; and (b) evaluate and synthesize the outcomes for these approaches for patients and families, healthcare providers, and health administrators/funders.

**Methods:**

This systematic scoping review will allow us to determine the extent, range, and nature of research activity related to initiatives designed to promote patient and family engagement in care. A comprehensive electronic literature search will be conducted in MEDLINE, EMBASE, and CINAHL. Studies will be included if they report on outcomes of a structured or systematic approach to the promotion of adult inpatient and family engagement in treatment in acute care settings. Studies will be selected in a two-stage screening process (title and abstract; full text) and quality will be assessed using the mixed methods assessment tool. Data extraction will include narrative descriptions of the intervention and classification of the behavior change techniques employed.

**Discussion:**

This review aims to identify and classify the specific behavior change techniques underpinning patient and family engagement interventions used in acute care hospital settings. By identifying the “active ingredients” in these interventions, our findings will be transferable to a wide range of acute care hospital contexts and populations.

**Electronic supplementary material:**

The online version of this article (10.1186/s13643-018-0694-9) contains supplementary material, which is available to authorized users.

## Background

Patient- and family-centered care is considered a key dimension of high-quality care [1] and has been increasingly linked to better health outcomes and lower use of health services [2–5]. It has been suggested, however, that adoption of the principles of patient-centered care focus fails to effect real change if patients themselves do not take active roles in the process [6].

Patient engagement involves patients and families taking on active roles within the healthcare system to improve health and healthcare services in collaboration and partnership with professionals [7, 8]. As Baker and colleagues note [8], patient engagement is founded on the premise that patients possess expertise related to healthcare by virtue of their own experiences. According to the Institute of Healthcare Improvement’s (IHI) 2017 Framework for Safe, Reliable and Effective Care [9], Patients in safe and reliable organizations, by capitalizing on the expertise of patients and their networks, effective patient engagement is seen to have numerous benefits to both the patient and the health care system, including reducing cost and waste [10]; enhancing system responsiveness to evolving user needs [10]; and promoting decision-making transparency and improving quality [10, 11].

The evidence base supporting the benefits of patient and family engagement is still evolving. While many healthcare systems have identified effective engagement of patients and families as a top priority, much remains to be learned about the nature and outcomes of approaches that seek to accomplish this goal. Systematic interventions that engage patients and families will often represent a change in usual practice for all stakeholders concerned, highlighting the importance of examining outcomes from diverse perspectives. Patient engagement initiatives are, by their nature, contingent upon multiple contextual factors, including the setting and whether the goal of the intervention is indirect or direct involvement [12]. Indirect engagement usually involves information gathering from the public to inform service delivery at a collective level without any role in decision-making, whereas direct engagement incorporates participation in decision-making, which can include treatment decisions made at an individual level [12]. Table 1 illustrates the ways in which direct engagement of patient and families intersect with the IHI framework’s domains of culture and learning systems.

**Table 1:** Intersection of the domains of the framework for safe, reliable and effective care and direct patient and family engagement (Adapted from Frankel et al., [9])

IHI domain	Patient and family engagement strategies
Leadership and accountability	Patients and the healthcare need to agree on goals and clearly define roles and accountability for goal achievement
	Clinical team members offer advice on clinical components
	Patients and families give their perspectives until there is agreement on goals
Teamwork and communication	Patients and families are considered part of the team and receive accurate, timely, and relevant communication about their health and care
Psychological safety	Patients should feel psychologically safe to share their concerns with the clinical team
	Healthcare team members receive patient perspectives openly and without judgment
	Patients are encouraged to be transparent about clinical signs, symptoms and treatment adherence
Negotiation	The healthcare team engages in collaborative negotiation with patients and families
	Healthcare team knows the patients’ priorities, worries, and desired outcomes
Transparency	Transparency with patients and families, particularly with respect to adverse events, is promoted
	Opportunities for patients and families to engage in care, making use of opportunities such as patient portals
Reliability	Patients can help to develop ways to make the process more reliable
Improvement and measurement	Patients share perspectives, experiences, and ideas about ongoing improvement efforts

Acute care hospitals are increasingly called upon to strengthen patient engagement efforts as a result of consumer demands and demographic shifts [13]. Effectively engaging patients and families during acute care hospitalizations has been associated with fewer adverse events [14], better self-management [15, 16], fewer diagnostic tests [17], decreased use of health services [18], and shorter lengths of stay in the hospital [19]. When patients and families are engaged in healthcare, there appears to be a greater likelihood that they can provide information essential to appropriate care planning [20], recognize errors in care delivery [21], and be more likely to adhere to treatment plans [22].

Wide variability in the implementation of practices designed to promote patient and family engagement in US hospitals was reported by Herrin and colleagues [5]. Their questionnaire included 37 distinct patient engagement practices appropriate for hospitals derived through a literature review and advice from an expert panel. Practices were classified into the following categories: a) organizational (e.g., formal policy for disclosing medical error); b) bedside (e.g., participation in shift change report); and c) access to information and shared decision-making (e.g., online access to personal health information). The most commonly reported barrier to implementing patient and family engagement strategies in this survey was “competing organization priorities” [5].

Approaches aimed at promoting patient and family engagement in treatment share the over-arching goal of changing behaviors of patients, families, and healthcare providers and possibly administrators. Patient engagement interventions are complex by nature and involve interacting components intended to produce changes in outcomes and behavior [23]. Patient and family engagement approaches simultaneously engage multiple actors (e.g., patients, families, healthcare providers, and healthcare leaders who sanction and often define the approach) and can occur in multiple and rapidly-changing contexts, particularly in an acute care hospital environment.

Understanding both the discrete elements of a complex intervention, as well as the underlying assumptions regarding why the intervention is hypothesized to be effective, serves to strengthen both the design of the intervention and the congruence between the intervention and the manner in which it is measured [24]. Behavior change techniques (BCTs) can be a key element of patient and family engagement approaches. This scoping review will contribute to the development of an evidence base detailing that BCTs have potential to be effective in patient and family engagement interventions.

BCTs are defined as “observable, replicable and irreducible component[s] of an intervention designed to alter or redirect causal processes that regulate behavior” [25]. In order to identify potential “active, effective” components within a specific approach to patient engagement, a reliable and systematic framework is essential [25]. Michie and colleagues have generated a taxonomy of 93 BCTs in 16 categories (see Table 2) that can be used to specify, interpret, and implement specific techniques used in patient and family engagement approaches. For example, within the category of goals and planning, goal setting, and problem solving are two techniques that may be used to promote patient and family engagement in treatment. Feedback on behavior may be provided to healthcare clinicians via patient experience evaluations of the engagement process.

**Table 2:** Overview of behavior change techniques [25]

BCT category	Example and definition
Goals and planning	*Problem solving*: analyze or prompt the person to analyze factors influencing the behavior and generate or select strategies that include overcoming barriers and/or increasing facilitators
Feedback and monitoring	*Feedback on behavior*: monitor and provide information on evaluative feedback
Social support	*Social support (practical):* advise on, arrange, or provide practical help for the performance of the behavior
Shaping knowledge	*Instruction on how to perform the behavior*: advise or agree on how to perform the behavior
Natural consequences	*Information about health consequences*: provide information (written, verbal, visual) about health consequences of performing the behavior
Comparison of behavior	*Information about others’ approval*: provide information about what other people think about the behavior
Associations	*Prompts/cues:* introduce or define environmental or social stimulus with the purpose of prompting or cueing the behavior.
Repetition and substitution	*Behavioral practice/rehearsal:* prompt practice or rehearsal of the behavior one or more times in a context or at a time when the performance may not be necessary in order to increase habit and skill
Comparison of outcomes	*Pros and cons:* advise the person to identify and compare reasons for wanting and not wanting to change the behavior
Reward and threat	*Social incentive*: inform that a verbal or non-verbal reward will be delivered if and only if there has been effort and/or progress in performing the behavior
Regulation	*Conserving mental resources:* advise on ways of minimizing demands on mental resources to facilitate behavior change
Antecedents	*Restructuring the social environment:* change or advise to change the social environment in order to facilitate performance of the wanted behavior or create barriers to the unwanted behavior
Identity	*Framing/re-framing:* suggest the deliberate adoption of a perspective or new perspective on behavior (e.g., its purpose) in order to change cognitions or emotions about performing the behavior
Scheduled consequences	*Situation-specific award:* arrange for reward following the behavior on one situation but not in another
Self-belief	*Verbal persuasion about capability:* tell the person that they can successfully perform the wanted behavior, arguing against self-doubts and asserting that they can and will succeed
Covert learning	*Vicarious consequences:* prompt observations of the consequences (including rewards and punishments) for others when they perform the behavior

## Objectives

This systematic scoping review focuses on synthesizing evidence relevant to the implementation of interventions designed to promote direct engagement of patients and families in care processes at an individual level. The specific objectives of this review are to:

- Identify and classify approaches used in acute care hospitals to engage patient and families in treatment according to the behavior change technique taxonomy [25]; and
- Evaluate and synthesize the outcomes for these approaches for patients and families, healthcare providers, and health administrators/funders.

## Methods

This systematic scoping review will allow us to determine the extent, range, and nature of research activity related to initiatives designed to promote patient and family engagement in care.

Guided by the PRISMA-P reporting guidelines [26], this review will include the following steps [27]: (a) identifying the research question, (b) identifying relevant studies, (c) study selection, (d) critical appraisal of studies, and (e) synthesizing and interpreting the results. Additional file 1 includes the completed PRISMA-P checklist. This protocol was not registered with PROSPERO.

### Identifying the research question

In collaboration with knowledge users from the provincial Health Quality Council and decision-makers from the Ministry of Health, the research questions for this review are (a) *“Which approaches to patient and family engagement in acute care hospitals have the most potential to result in positive outcomes for: patients and families; health care providers; health systems and administrators/funders?”* and (b) “*Which behavior change techniques can be effectively implemented to promote effective patient and family engagement?”*

### Identifying relevant studies

We recognize that the type of evidence sought requires use of a broad range of potential sources, including peer-reviewed academic publications and associated reference lists as well as gray literature such as non-peer-reviewed studies, and online reports. The New York Academy of Medicine [28] includes the following documents in their definition of gray literature: reports (pre-prints, preliminary progress and advanced reports, technical reports, statistical reports, memoranda, state-of-the art reports, market research reports etc.), theses, conference proceedings, technical specifications and standards, bibliographies, and official documents not published commercially such as government reports.

A comprehensive electronic literature search will be conducted by an experienced medical librarian (EW) in MEDLINE, EMBASE, and CINAHL from inception to the date we will conduct our most recent updated search. Our search strategy will include the following key terms and synonyms: acute care; hospitals; caregivers; family; and patient participation, empowerment, engagement or involvement. Please see Additional file 2 for the comprehensive search strategy in MEDLINE. An English language publication limit will be used. The reference lists of relevant studies will be examined to identify other relevant articles.

### Study selection

Literature search results will be uploaded into Covidence™ Systematic Review Software [29] after removing duplicate references. This software provides a decision dashboard and annotation tool, as well as the capacity to create forms for screening and extracting data. Additional duplicates missed by the reference software will be removed as identified. Studies will be selected in two phases: (a) title and abstract screening and (b) full-text screening/review.

Inclusion and exclusion criteria were developed based upon a preliminary literature review and the advice of knowledge users and decision-makers. In order to be included in this scoping review, the studies must (a) have taken place within an acute care hospital setting (including inpatient rehabilitation), (b) describe or include a structured or systematic approach to promotion of patient and/or family participation in treatment, which could include organizational practices, bedside practices or access to information practices, (d) adults populations only, and (d) describe the outcomes of the interventions from any one of the following stakeholder perspectives: patients and families; health care providers; health systems; or administrators/funders. We will include studies published in English for this scoping review.

We will exclude papers addressing patient engagement initiatives in the following populations: children and adolescents; community or home settings; oncology patients (because this group often experiences rapid transitions between community, outpatient and inpatient settings) and emergency department settings. We will also exclude papers focused upon patient participation in research, databases, quality improvement (e.g., patient advisory councils) or healthcare service re-design, patient needs or knowledge assessments, patient activation studies which lack specific interventions designed to promote patient, and family engagement.

The review team is comprised of ten researchers, including one patient representative who possesses an academic background in a non-health-related discipline. The GRIPP2-SF reporting checklist [30] provides guidance for reporting PPI in research in five areas. In this scoping review, the aim of including a patient representative is to ensure that the engagement initiatives selected for inclusion are considered potentially useful from the patient’s perspective. The patient representative participated in the initial identification of the research question and will be a full partner in all aspects of this scoping review. We will report on the outcomes of PPI in terms of the manner in which including PPI influenced the outcomes of this scoping review.

### Title and abstract screening

Team training sessions will consist of group screening of 30–50 titles. The inclusion and exclusion criteria will be pilot-tested to review and revise the final set of eligibility criteria and to enhance the screening reliability of team members. Titles and abstracts will be screened by two reviewers.

### Full-text screening and review

Two researchers will independently review each of the full-text articles to ensure the inclusion criteria have been met. Discrepancies will be discussed between the researchers to achieve consensus and disputes resolved by an arbitrator (EP).

### Data extraction

A standard data extraction form will be prepared and pilot-tested prior to data extraction (see Additional File 3). Data will be extracted on the key characteristics of included studies. These will include information related to (a) study identification (author, year of publication, journal, country), (b) study design (methodological approach, eligibility criteria, sample size, data sources), (c) setting characteristics (services provided, numbers of hospital beds, funding model), (d) patient and family characteristics (age, diagnoses, sex), (e) description of intervention (narrative description, BCT category and specific BCT), and (f) reported outcomes for patients and families, healthcare providers, healthcare systems administrators, or funders. Coding of BCT categories and techniques will be facilitated through use of the BCTs Taxonomy app [31].

### Critical appraisal of studies

Given our preliminary literature search, we anticipate identifying primarily descriptive, qualitative small-scale studies with a smaller number of mixed methods or quantitative studies. All included studies will be critically evaluated using the mixed methods appraisal tool [32]. This tool is designed to facilitate complex systematic reviews by allowing for the concurrent appraisal, description and scoring of mixed methods, qualitative and quantitative studies. For each study, an overall methodological quality score ranging from (* one criterion met to **** all criteria met) will be calculated. Mixed methods studies are ranked by the weakest component (either qualitative or quantitative). As needed, study authors will be contacted for additional information as is usual when reviewing primary studies.

### Synthesizing and interpreting the results

Narrative synthesis will be conducted in three stages [33]: free line-by-line coding of the findings of the primary studies; categorization of the free codes into related topics to develop descriptive themes; and the construction of analytical themes that can adequately describe and explain the descriptive themes. We will link the descriptive themes, including nature of the intervention and the BCTs employed, with the outcomes reported. By generating a series of hypotheses regarding the ways in which patient engagement interventions may impact outcomes in acute care settings, we will inform the design of future research in this area.

All evidence will be included and the strength of the evidence rated for each of the patient engagement approaches and BCTs identified, following Wranik and colleagues’ [27] categories (Table 3).

**Table 3:** Evidence ratings [27]

Strong evidence of effect	More than one study with a MMAT rating of ****
Moderate evidence of effect	One or more studies with a MMAT of ***
Limited evidence of effect	One or more studies with a MMAT rating of ** or less
Conflicting evidence	Inconsistent findings across studies, with MMAT ratings of *** or more
No evidence	No studies, or conflicting findings with MMAT ratings of ** or less

*Overall methodological rating (*one criterion met to ****all criteria met)

### Discussion

This review aims to not merely identify, but to also classify the “active ingredients” underpinning the approaches used in acute care hospitals to engage patient and families in treatment by using the behavior change technique taxonomy [25]. Specification of the BCTs that have resulted in positive outcomes for patients and families, healthcare providers, health systems, or health administrators/funders maximizes the transferability of our findings to a wide range of acute care contexts. The findings generated through this synthesis will provide an evidentiary basis for the development of, and future research related to, tailored approaches to patient and family engagement interventions.

A key potential challenge in this review may be incomplete reporting of key aspects of the patient and family engagement approach in the included literature. This challenge will be mitigated by contacting study authors as required to access additional information needed to classify the BCTs used in the intervention described in the study. A limitation of this review is recognized to be the exclusion of databases, such as Applied Social Sciences Index and Abstracts and PsycINFO, which may potentially contain additional relevant articles. Future systematic reviews on patient engagement may choose to include these databases, and further include the strategy of forward searching.

While patient engagement is increasingly seen as a cornerstone of health system re-design, the value of this approach is not uncontested. Concerns about tokenism, sub-optimal quality of patient involvement and insufficient resources for meaningful engagement of patients [34], as well as power imbalances affecting the meaningful implementation of patient engagement [35], are concerns warranting ongoing attention in these efforts. The term “patient engagement” itself [36] suffers from inconsistencies in definition. As an emerging focus of research, significant work is still required to advance the evidence base.

This review will result in a synthesis of the evidence relating to interventions designed to promote patient and family engagement in treatment in acute care hospital settings. By describing the nature of these interventions and identifying the BCTs underpinning successful interventions, the findings of this review will produce transferable findings that can be applied to the development and evaluation of patient and family engagement strategies.

## Additional files

Additional file 1: PRISMA-P Checklist. The data provided shows a completed copy of the PRISMA-P Checklist to guide readers in assessing the quality of the current protocol. (DOCX 29 kb)

Additional file 2: Search Strategy. Comprehensive Medline strategy. (DOCX 23 kb)

Additional file 3: Draft Data Extraction Tool. (DOCX 18 kb)

## Abbreviation

BCTs: Behavior change techniques
