# Supplementary material for: Structured approaches to promote patient and family engagement in treatment in acute care hospital settings: protocol for a systematic scoping review
Source: Syst Rev. 2018 Feb 26;7:35. doi: 10.1186/s13643-018-0694-9 (PMC5827976; doi:10.1186/s13643-018-0694-9)
Supplement: Supplementary file 2 — Search Strategy. Comprehensive Medline strategy. (DOCX 23 kb) [file 13643_2018_694_MOESM2_ESM.docx]

Additional file 2: Search Strategy. Comprehensive Medline strategy.

Database(s): Ovid MEDLINE(R) In-Process & Other Non-Indexed Citations and Ovid MEDLINE(R) 1946 to Present
Search Strategy:

| **#** | **Searches** | **Results** |
| --- | --- | --- |
| 1 | acute care.mp. | 17713 |
| 2 | hospitals/ or exp hospitals, community/ or exp hospitals, general/ or exp hospitals, group practice/ or exp hospitals, high-volume/ or exp hospitals, low-volume/ or exp hospitals, private/ or exp hospitals, public/ or exp hospitals, rural/ or exp hospitals, satellite/ or exp hospitals, teaching/ or exp hospitals, urban/ or secondary care centers/ or tertiary care centers/ | 197791 |
| 3 | hospital*.mp. | 1356031 |
| 4 | inpatients/ | 17400 |
| 5 | (in-patient? or inpatient?).mp. [mp=title, abstract, original title, name of substance word, subject heading word, keyword heading word, protocol supplementary concept word, rare disease supplementary concept word, unique identifier] | 1503794 |
| 6 | or/1-5 | 2652901 |
| 7 | patient participation/ | 22552 |
| 8 | caregivers/ | 29583 |
| 9 | family/ | 72856 |
| 10 | patients/ | 19652 |
| 11 | 8 or 9 or 10 | 116627 |
| 12 | consumer participation/ | 16322 |
| 13 | 11 and 12 | 412 |
| 14 | ((carer? or caregiver? or client? or consumer? or families or family or patient? or stakeholder? or user?) adj2 (empower* or engage* or participat*)).ab. /freq=2 | 3077 |
| 15 | ((carer? or caregiver? or client? or consumer? or families or family or patient? or stakeholder? or user?) adj2 (empower* or engage* or participat*)).ti. | 2943 |
| 16 | ((carer? or caregiver? or client? or consumer? or families or family or patient? or stakeholder? or user?) adj involve*).ab. /freq=2 | 980 |
| 17 | ((carer? or caregiver? or client? or consumer? or families or family or patient? or stakeholder? or user?) adj involve*).ti. | 1136 |
| 18 | ((carer? or caregiver? or client? or consumer? or families or family or patient? or stakeholder? or user?) adj2 (empower* or engage* or participat*)).kf. | 752 |
| 19 | ((carer? or caregiver? or client? or consumer? or families or family or patient? or stakeholder? or user?) adj involve*).kf. | 305 |
| 20 | or/14-19 | 7600 |
| 21 | 7 or 13 or 20 | 28535 |
| 22 | 6 and 21 | 5688 |
| 23 | limit 22 to English | 5261 |
| 24 | remove duplicates from 23 | 4773 |
