# Supplementary material for: Structured approaches to promote patient and family engagement in treatment in acute care hospital settings: protocol for a systematic scoping review
Source: Syst Rev. 2018 Feb 26;7:35. doi: 10.1186/s13643-018-0694-9 (PMC5827976; doi:10.1186/s13643-018-0694-9)
Supplement: Supplementary file 3 — Draft Data Extraction Tool. (DOCX 18 kb) [file 13643_2018_694_MOESM3_ESM.docx]

Scoping Review Data Extraction Sheet

| Primary author/organization: | | |
| --- | --- | --- |
| Title of article: | | |
| Source of publication (Name of journal or report): | | |
| Year of publication: | | |
| Reviewer initials: | | |
| Sponsorship/Funding | |  |
| Country | |  |
| Inclusion Criteria | |  |
| Type of Patient Engagement Program | |  |
| Describe the Intervention | |  |
| Duration of Program | |  |
| Theoretical Framework  (Identify and describe, if present) | |  |
| Study Design (Quantitative) | Case Series |  |
|  | Cross-Sectional (Pre- and post) |  |
|  | Case-control |  |
|  | Retrospective Cohort |  |
|  | Prospective Cohort |  |
|  | RCT |  |
| Study Design (Qualitative) | Basic Interpretive |  |
|  | Phenomenological |  |
|  | Grounded Theory |  |
|  | Ethnographic |  |
|  | Case Study |  |
| Study Design (Mixed Methods) | QUAL core  QUAN core  Sequence |  |
| Non-Research Document | Describe type |  |
| Type of Hospital | Teaching |  |
|  | Community |  |
|  | Rehabilitation |  |
|  | Psychiatric/Mental Health |  |
| Type of Unit | |  |
| Patient Engagement Program Characteristics | |  |
|  | Aims and Purpose |  |
|  | Description of Intervention |  |
|  | BCTs Used: Category |  |
|  | BCTs Used: Techniques |  |
|  | Duration of Intervention |  |
| Participants | Number of participants |  |
|  | Medical diagnosis |  |
|  | Age range |  |
|  | Inclusion criteria (patients) |  |
|  | Exclusion criteria (patients) |  |
|  | Inclusion of family/informal caregivers |  |
|  | Inclusion of health care providers |  |
| Methods | Type of study |  |
|  | Instruments Used |  |
| Results | Patient outcomes |  |
|  | Health care provider outcomes |  |
|  | Health system & effectiveness outcomes |  |
|  | Funder outcomes |  |
| Comments | |  |
